# Vertical contact forces affect vibration perception in human hairy skin

**DOI:** 10.7717/peerj.15952

**Published:** 2023-09-04

**Authors:** Daniel Schmidt, Guenther Schlee, Thomas L. Milani, Andresa M. C. Germano

**Affiliations:** 1 Motor Control, Cognition, and Neurophysiology, Faculty of Behavioral and Social Sciences, Institute of Human Movement Science and Health, Chemnitz University of Technology, Chemnitz, Saxony, Germany; 2 Biophysics and Human Performance Lab, W.L. Gore and Associates, Putzbrunn, Bavaria, Germany; 3 Department of Human Locomotion, Faculty of Behavioral and Social Sciences, Institute of Human Movement Science and Health, Chemnitz, Saxony, Germany

**Keywords:** Vibration perception thresholds, Hairy skin, Cutaneous sensitivity, Contactor force, Sensitivity, VPTs

## Abstract

**Background:**

Skin is the largest organ of the human body and fulfills many important functions, like detecting mechanical stimuli. Skin can be divided into glabrous (non-hairy) and hairy skin. These two skin types differ with regard to their mechanical properties and in the distribution of mechanoreceptors. Although many investigations focus on glabrous skin, hairy skin still plays a fundamental role in various activities, *e.g*., with regard to the perception of pleasantness or for developing wearable vibrotactile devices for pattern recognition in persons with disabilities. Unfortunately, investigations on influencing factors, like vertical contactor force, are scarce for hairy skin. Similarly, it would also be interesting to investigate whether regional vibratory sensitivity differences are present across the human torso. Hence, this study investigated the effects of vertical contactor forces and different anatomical locations on vibration perception. Four anatomical torso regions were studied. Based on findings in glabrous skin, we generally hypothesized improved vibration perception with increasing contactor forces and regional sensitivity differences between the anatomical locations.

**Methods:**

Forty young and healthy individuals participated (23.0 ± 2.0 yrs), and vibration perception thresholds (VPTs) were determined at 30 Hz for three vertical force levels (0.6, 2.4, and 4.8 N) at four torso locations (sternum, deltoid/shoulder, lower back, middle lateral torso side).

**Results:**

Higher contactor forces resulted in lower VPTs corresponding to improved vibration perception, regardless of anatomical location. In addition, the sternum region was more sensitive than the remaining three regions, regardless of force level. The reasons for these findings may be a varying number and activation pattern of afferents activated under the different conditions. The findings of this study complement the understanding of vibrotactile sensitivity in hairy skin and may offer implications when developing vibrotactile devices or clothing/textiles, for example.

## Introduction

Of all organs in the human body, skin is considered the largest, fulfilling many highly important physiological tasks. These include preventing the intrusion of microorganisms and providing protecting from dehydration (Firooz et al., 2012), as well as detecting mechanical stimuli (Holowka et al., 2019). Skin can be divided into non-hairy (glabrous) and hairy skin. The latter covers the vast majority of the body surface (approx. 90%; Zimmerman, Bai & Ginty, 2014). Glabrous skin mechanoreceptors, particularly in the foot sole and finger tips, are involved in human balance regulation (Germano, Schmidt & Milani, 2016) and explorative and precision tasks (Birznieks et al., 2009; Westling & Johansson, 1987).

Hairy skin exhibits numerous differences compared to glabrous skin in terms of mechanical properties, such as a lower stiffness (Hamalainen, Warren & Gardner, 1985; Pubols, 1982). Regarding sensory (mechano) receptors, hairy skin does not contain Meissner corpuscles (Munge & Ide, 1988; Corniani & Saal, 2020), but instead hair follicle receptors and associated afferents (Mahns et al., 2006; Verrillo, 1977). In addition, SA (slowly adapting) type I units (associated with touch domes), SAII units (usually associated with Ruffini corpuscles), and several other FA (fast adapting) units, such as field units and Vater-Pacini corpuscles (Corniani & Saal, 2020), reside in hairy skin. In terms of its physiological-functional role, hairy skin is less involved in explorative and precision tasks (Edin & Abbs, 1991). Generally, hairy skin is considered to be less sensitive than glabrous skin (Cassella, Ashford & Kavanagh-Sharp, 2000; Gregg, 1951; Lamoré & Keemink, 1988; Zimmerman, Bai & Ginty, 2014), likely due to a lower density of several afferent units (Edin & Abbs, 1991; Lamoré & Keemink, 1988), especially with regard to mechanical stimuli (*e.g*., skin touch or vibrations).

However, there are scenarios in which the hairy skin exceeds physiological capabilities of glabrous skin: mechanosensitive afferents from hairy skin seem to play a more dominant role in the perception of pleasantness (Nagi et al., 2015; Watkins et al., 2021), indicating a role in socially relevant tasks (Olausson et al., 2010). In rodents, it was even proposed that these afferents play a role in generating hormonal responses (Uvanas-Moberg, Arn & Magnusson, 2005). Additionally, hairy skin contains more slowly adapting and unmyelinated units compared to glabrous skin (Järvilehto, Hämäläinen & Soininen, 1981; Watkins et al., 2021). Hairy skin low-threshold mechanoreceptors were shown to not only play an important role in the perception of finger movements, but also to precede proprioceptive muscle afferents (Edin & Johansson, 1995). Edin & Johansson (1995) demonstrated that under certain circumstances, cutaneous afferents “overwrite” information from muscle spindles when assessing finger posture. Another study also showed that cutaneous afferents might even outperform the dynamic velocity sensitivity of muscle spindle afferents (Grill & Hallett, 1995). Furthermore, when air puffs are applied to the skin, hairy skin exhibits equal or greater sensitivity characteristics than glabrous skin (Hamalainen, Warren & Gardner, 1985). It becomes evident that research on hairy skin is indeed an interesting and relevant field. Results from such research could, for example, help develop wearable vibrotactile (torso) devices for pattern recognition in people with disabilities (Jones, Lockyer & Piateski, 2006; Seim et al., 2022), or when manufacturing textiles/clothes.

When assessing vibrotactile cutaneous sensitivity, several influencing factors should be considered. One of these is the vertical contactor force with which the probe vibrates on the skin. Many studies (mostly conducted on glabrous skin) showed improved perception with increasing contactor forces (Cassella, Ashford & Kavanagh-Sharp, 2000; Lowenthal, Derek & Hockaday, 1987; Zippenfennig, Wynands & Milani, 2021). Unfortunately, studies usually apply quite different contactor forces, making comparability and interpretation of results difficult. Additionally, these studies were mostly conducted on glabrous skin and/or at only a few anatomical locations. Hence, there is generally only a limited number of studies examining these aspects in different anatomical regions. Furthermore, it would be interesting to investigate potential regional sensitivity differences across the torso, since the literature to date is not definitive (Schmidt et al., 2020; Stronks, Parker & Barnes, 2016). One significant contributor to skin sensitivity is the afferent innervation density of the corresponding regions, which can be assessed by microneurographic readings (Corniani & Saal, 2020). Interestingly, such studies are lacking at various body trunk regions (Corniani & Saal, 2020). In addition, there are only a few studies investigating regional vibratory sensitivity differences within the torso and those exhibit certain limitations. For example, Wilska (1954)—although investigating many different body regions—only included one male participant and did not report vertical contactor forces or skin temperatures. Another study focusing on vibratory spatial acuity only included four participants (one female) (Cholewiak, Brill & Schwab, 2004). Furthermore, the same study assessed vibratory spatial acuity horizontally around the abdomen at the navel region and not regional differences of absolute vibratory sensitivity.

Such knowledge would also serve industrial branches, such as the development of vibrotactile devices in the disabled population, or the clothing industry. Therefore, the present study investigated the effects of vertical contactor forces and anatomical locations on vibration perception at four hairy skin locations of the human torso. We hypothesized an improved vibration perception with increasing vertical contactor forces and corresponding regional sensitivity differences between anatomical locations. Although dealing with vibration perception at glabrous skin areas, the majority of the literature (*e.g*., Schlee, Sterzing & Milani, 2009; Germano et al., 2016; Schmidt, Germano & Milani, 2017, 2018; Schmidt et al., 2020) showed no sex difference in young adults. Therefore, we hypothesized no sex differences for anatomical location or force level in the present study.

## Materials and Methods

Forty young individuals (20 females, 20 males) participated in this study. Anthropometric data is presented in Table 1. Participants were healthy and had no neurological disorders potentially affecting vibration perception thresholds (VPTs), such as diabetes mellitus or (poly-)neuropathy. Furthermore, they had no visible irritation or inflammation of the measured skin areas (determined by visual inspection) and were not taking any medication that could alter the perception of sensitivity. Prior to data collection, all recruited individuals were informed about the content and aim of this study, and provided their written informed consent. The present study was conducted at the Chemnitz University of Technology and in accordance with the recommendations of the Declaration of Helsinki. Furthermore, this study was approved by the Ethics Committee of the Chemnitz University of Technology (ID: V-277-17-DS-KUS/WUS-22062018).

**Table 1:** Anthropometric data.

Mean ± SD	Age (yrs)	Height (cm)	Body mass (kg)
All (*n* = 40)	23.0 ± 2.0	176.3 ± 9.6	73.6 ± 10.1
Females (*n* = 20)	23.0 ± 2.0	170.5 ± 7.2	68.6 ± 10.5
Males (*n* = 20)	23.0 ± 2.1	182.0 ± 8.3	78.5 ± 6.8

**Note:**

Anthropometric data of the participants. Numbers represent means ± standard deviations (SD).

To assess VPTs, vibration stimuli with a frequency of 30 Hz were delivered using a modified vibration exciter (Typ 4180; Brüel & Kjaer Vibro GmbH, Darmstadt, Germany) supplied by a powerbank (XTPower MP-3200; Batteries and Power Solutions GmbH, Ellwangen, Germany). Thirty Hertz were chosen because there is no audible tone (in contrast to high-frequency vibrations, where participants would potentially be distracted). Furthermore, we expect superficial mechanoreceptors to be particularly affected when wearing shirts and other clothing. There are mechanoreceptors located superficially below the skin, which are responsive at 30 Hz, such as touch domes (Handler & Ginty, 2021) or hair follicle sensors (Bolanowski, Gescheider & Verrillo, 1994). This constituted another reason to test at 30 Hz. The perceived vibration amplitude (µm) constituted the target parameter. During each trial, the acceleration of the vibrating plastic contactor (7.8 mm diameter with rounded edges and no surround) was measured using an integrated acceleration sensor (MMA2240KEG; NXP Semiconductors Netherlands B.V., 141 Eindhoven, The Netherlands). This acceleration signal was then filtered and double-integrated to acquire the vibration amplitude. We ensured that the measurement and integration of the acceleration signal accurately reflected the actual amplitude of the plastic contactor. This was achieved by calibrating and verifying the entire system using a high-precision capacitive position sensor (CS05; Micro-Epsilon Messtechnik GmbH & Co. KG, Ortenburg, Germany) during the manufacturing process of the device. The vibrating device was freely moveable in all directions, which allowed the contactor to be aligned perpendicularly to the anatomical locations. In addition, a one-dimensional force sensor (DS050A9; disynet GmbH, BrüggenBracht, Germany) was used to display the vertical contactor force on the skin. During the measurements, the absolute permissible variation of vertical contact force was ±0.3 N. A narrower variation range was not possible due to slight movements of the participants (*e.g*., breathing).

Since skin temperature affects cutaneous sensitivity (Germano, Schmidt & Milani, 2016; Schlee, Sterzing & Milani, 2009), the temperature of the measured anatomical locations was monitored using a non-contact infrared thermometer (UNI-T UT301C; Batronix GmbH & Co. KG, Preetz, Germany). Skin temperatures were measured for each anatomical location before (pre) and after (post) all corresponding trials: three vertical force levels with three VPT values, each (details below). Following the regulations from EN ISO/IEC 17025, room temperatures were also monitored (digital C28 type K thermocouple; Comark Instruments, Norwich, England, UK) and kept within a range of 23.0 ± 2.0 °C.

During data collection, all participants wore noise-cancelling headphones (QuietComfort 25 Acoustic Noise Cancelling headphones, Bose Corp., Framingham, MA, USA) to prevent distraction caused by external noise sources.

**Figure 1:**
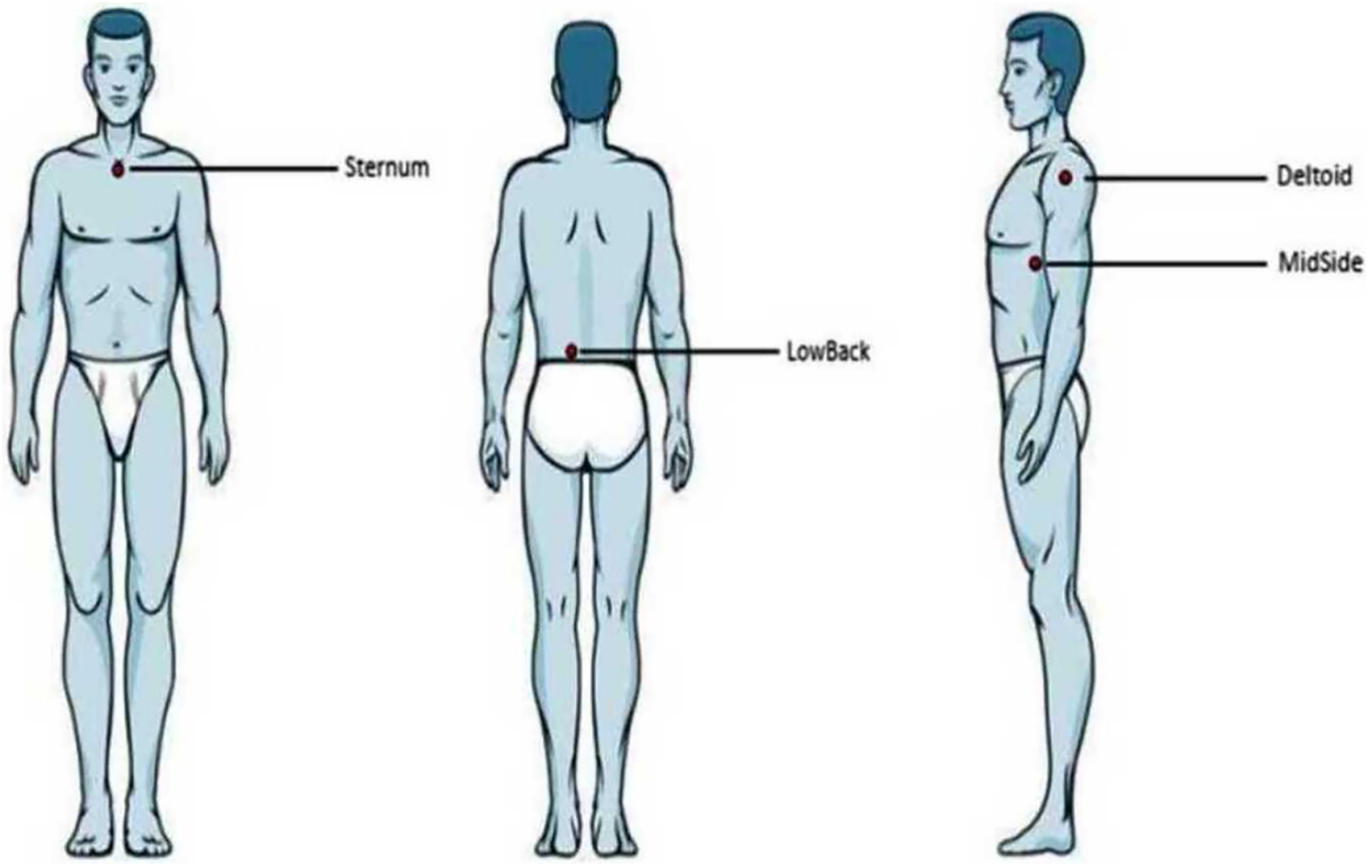
Anatomical locations. Illustration of the four anatomical locations examined. From left to right: anterior, posterior, and lateral view.

During testing, the participants first had an acclimatization period of 10 min to adapt to the room temperature and to avoid large-scaled fluctuations of skin temperature. Four anatomical locations of the human torso were manually palpated, located, and marked with a pen: the sternum (the upper region approx. 2 cm below the occurrence of the bony tissue), the middle region of the lower back just next to the spine (LowBack, the region coming from the iliac crests toward the spine, approx. 3 cm lateral to the spine), the middle, lateral aspect of the torso (MidSide, the middle aspect of the torso side by incorporating half of the proximal-distal distance and half of the anterior-posterior distance), and the skin covering the deltoid muscle (Deltoid, middle region of the palpated muscle tissue) (Fig. 1). Non-centric anatomical positions (Deltoid, LowBack, MidSide) were tested in a randomized fashion for the left or right side of the body (not both sides). Since Hamalainen, Warren & Gardner (1985) found an effect on skin sensitivity due to shaving the hair, the participants in our study were not shaved.

For each anatomical location, VPTs were assessed at three different vertical force levels: 0.6, 2.4, and 4.8 N. This corresponds to a pressure of 1.26, 5.02, and 10.04 N/cm^2^, respectively. Participants did not report pain in any of the three force levels. Following an earlier investigation (Zippenfennig, Wynands & Milani, 2021), we selected these force levels to improve comparability (identical contactor dimensions). Furthermore, unpublished data from the apparel industry indicates that, when wearing base layers, pressures are comparable to the lowest force level used in this study. For each vertical force level, three individual VPTs were assessed. This resulted in a total of four (anatomical locations) × three (force levels) × three (VPTs) = 36 trials for each participant. These VPT trials were randomized as follows: anatomical locations were randomized. For each location, the force levels were then randomized. Subsequently, once a certain force level was selected, the three individual VPT trials were carried out consecutively. Sufficient pause lengths between those three trials were guaranteed (based on participant feedback), especially following higher force levels.

**Figure 2:**
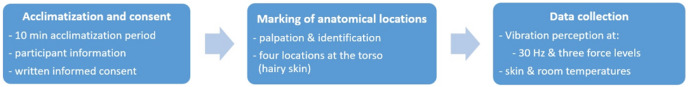
Workflow diagram. Workflow diagram to highlight the three major steps (Acclimatization and consent, Marking of anatomical locations, Data collection) within the protocol of this study.

**Figure 3:**
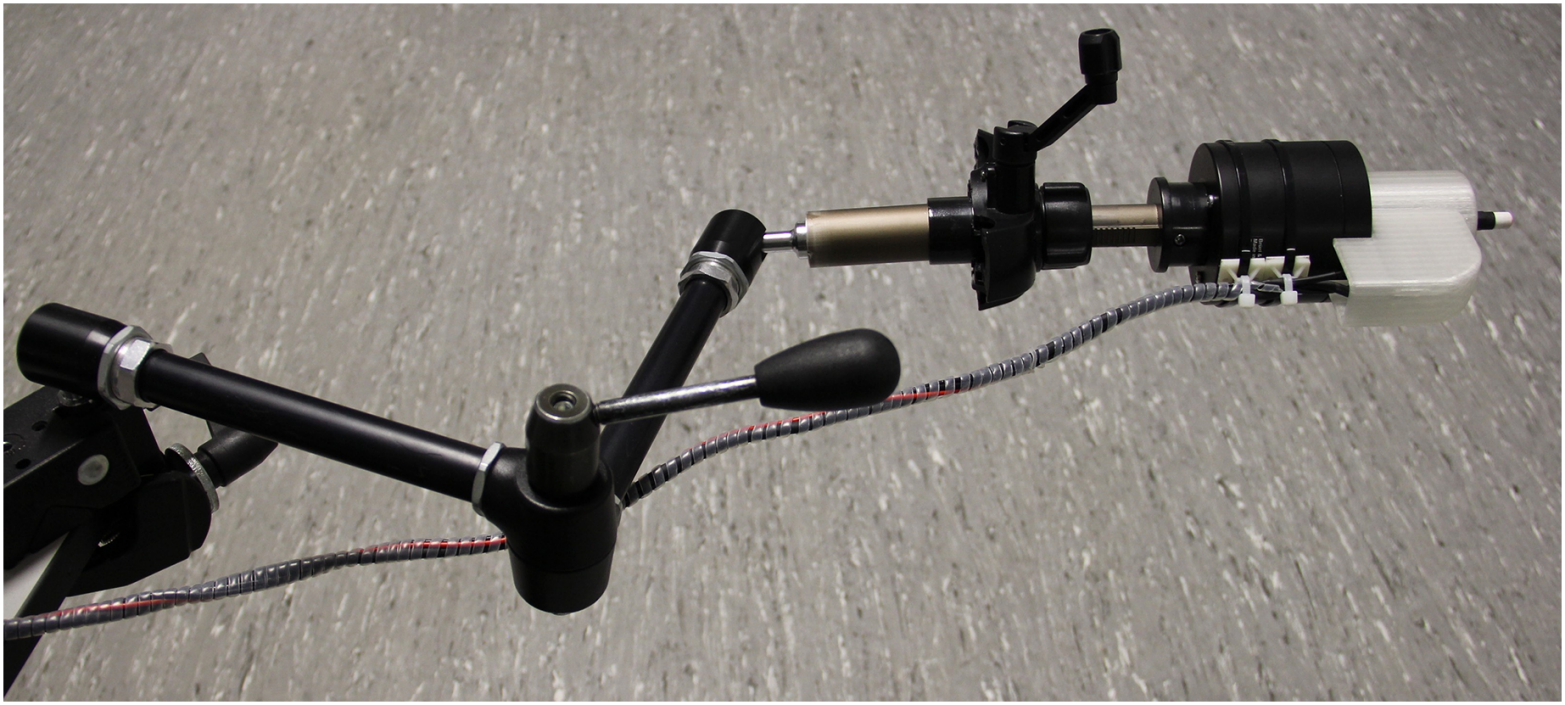
Vibration exciter. Photograph of the setup. An adjustable mechanical arm allowed a threedimensional positioning of the vibration exciter and contactor (white tip on the right side of the picture). A one-dimensional force sensor was integrated into the setup to measure contact forces.

VPTs were assessed similarly to a protocol introduced by Mildren, Strzalkowski & Bent (2016) and as described in earlier work from our group (Schmidt et al., 2020). In brief, participants held a trigger in their hand and were instructed to push the button as soon as they perceived a vibration. Sinusoidal vibration stimuli (30 Hz) were delivered at a duration of 2.0 s with randomized pause lengths in between the individual ascending and descending bursts ranging between 0.5 and 5.0 s. The first vibration stimulus was supra-threshold and could easily be perceived (amplitude 102.4 µm, consistent for all participants and trials). The subsequent bursts were set at 50% of the amplitude of the previous burst that was clearly perceived. If a stimulus was not detected, the amplitude was set at an intermediate level between the unperceived stimulus and the last perceived stimulus. VPTs were determined as the lowest perceived amplitude within the iterations (in most cases 11 iterations, with four iterations following the second stimulus that was not perceived). On average, one single VPT trial took about 60 s. The protocol also included catch stimuli (duration 4–7 s) to make sure participants did not press the trigger when there was no vibration stimulus. No such false positive trials were evident. In addition, the temperature of each of the four measured anatomical locations was monitored before and after completing the entire VPT test procedure. An illustration of the overall workflow and setup is provided in Figs. 2 and 3, respectively.

Medians and median absolute deviations were calculated from the three individual VPT trials. The Shapiro-Wilk test was used to test data distribution (α = 0.05). To test for differences between sexes, the Mann-Whitney-U test or the independent t-test were used, depending on the results of the Shapiro-Wilk test (α = 0.05, respect.). Pairwise differences between the three force levels were investigated using the Wilcoxon signed rank test or the dependent t-test. Due to the number of force levels, the level of significance was corrected from α = 0.05 to α = 0.05/3 = 0.017. Again, the Wilcoxon signed rank test or the dependent t-test were used to detect differences between the four anatomical locations for each force level. Since four anatomical locations were examined, alpha was corrected from α = 0.05 to α = 0.05/4 = 0.0125. Effect sizes were calculated using Cohen’s D and Vargha and Delaneys’s A.

Since the majority of VPT data was heteroscedastic, and logarithmizing the raw data resulted in an elimination/reduction of heteroscedasticity, all inferential statistical tests and effect size calculations were conducted on logarithmized data. This approach is based on recommendations from an early study (Nevill & Atkinson, 1997). In a more recent work, we also relate this approach to VPT data and the interested reader is referred to Schmidt, Germano & Milani (2019).

## Results

Results are presented in either tables or boxplots. Tables show mean ± standard deviation (SD) values calculated based on the median values from the individual three VPT trials. For the boxplots, the horizontal line within each of the boxes represents the median and the boxes represent the interquartile range (IQR, 25th to 75th percentile). The lower and upper whiskers (25th percentile − (1.5 × IQR) and 75th percentile + (1.5 × IQR), resp.) do not contain outliers, these are not depicted in the boxplots.

Due to an offset error, skin temperature data was only analyzed for 18 participants and mean ± SD temperatures were as follows (pre/post, resp.): sternum 34.0 ± 0.9/33.7 ± 0.8 °C; low back: 33.9 ± 1.1 °C/33.6 ± 0.9 °C; middle region of lateral torso: 33.2 ± 1.1 °C/33.3 ± 0.7 °C; and deltoid region: 32.1 ± 1.0 °C/32.8 ± 1.2 °C. Significant differences between pre and post skin temperatures were only evident for the deltoid region (dependent t-test: *p* = 0.033, α = 0.05).

For each location and each vertical force level, no significant differences were detected between the VPTs of female and male participants (Table 2). Hence, all further analyses were conducted with pooled data from both sexes.

**Table 2:** Sex comparisons of vibration perception thresholds (VPTs).

		Sternum		LowBack		MidSide		Deltoid	
Mean ± SD VPTs (µm)		Female	Male	Female	Male	Female	Male	Female	Male
Force levels	**0.6 N**	21.8 ± 18.0	19.9 ± 15.0	28.7 ± 11.3	34.0 ± 29.4	28.4 ± 10.2	36.7 ± 20.1	32.1 ± 13.1	37.2 ± 12.9
	*p*-values	0.782^a^		0.659^b^		0.349^a^		0.402^b^	
	Effect sizes	0.088^I^		0.543^II^		0.300^I^		0.579^II^	
	**2.4 N**	10.0 ± 3.3	10.3 ± 5.6	21.7 ± 9.0	23.0 ± 12.1	14.9 ± 5.3	17.0 ± 8.6	17.0 ± 8.9	23.1 ± 12.7
	*p*-values	0.774^a^		0.754^a^		0.719^a^		0.076^a^	
	Effect sizes	0.094^I^		0.100^I^		0.115^I^		0.577^I^	
	**4.8 N**	8.2 ± 2.5	8.7 ± 3.2	15.9 ± 6.0	19.2 ± 7.2	10.7 ± 2.6	12.9 ± 5.5	13.2 ± 7.0	16.1 ± 7.9
	*p*-values	0.755^a^		0.2016^a^		0.265^a^		0.195^a^	
	Effect sizes	0.099^I^		0.411^I^		0.360^I^		0.417^I^	

**Notes:**

a Independent t test.

b Mann-Whitney-U test.

I Cohen’s D.

II Vargha and Delaney’s A.

Mean ± SD VPTs at 30 Hz for both sexes, all locations, and all force levels (0.6, 2.4, and 4.8 N) (raw data). No significant differences were found (inferential statistical tests and effect size calculations were performed on logarithmized data).

**Figure 4:**
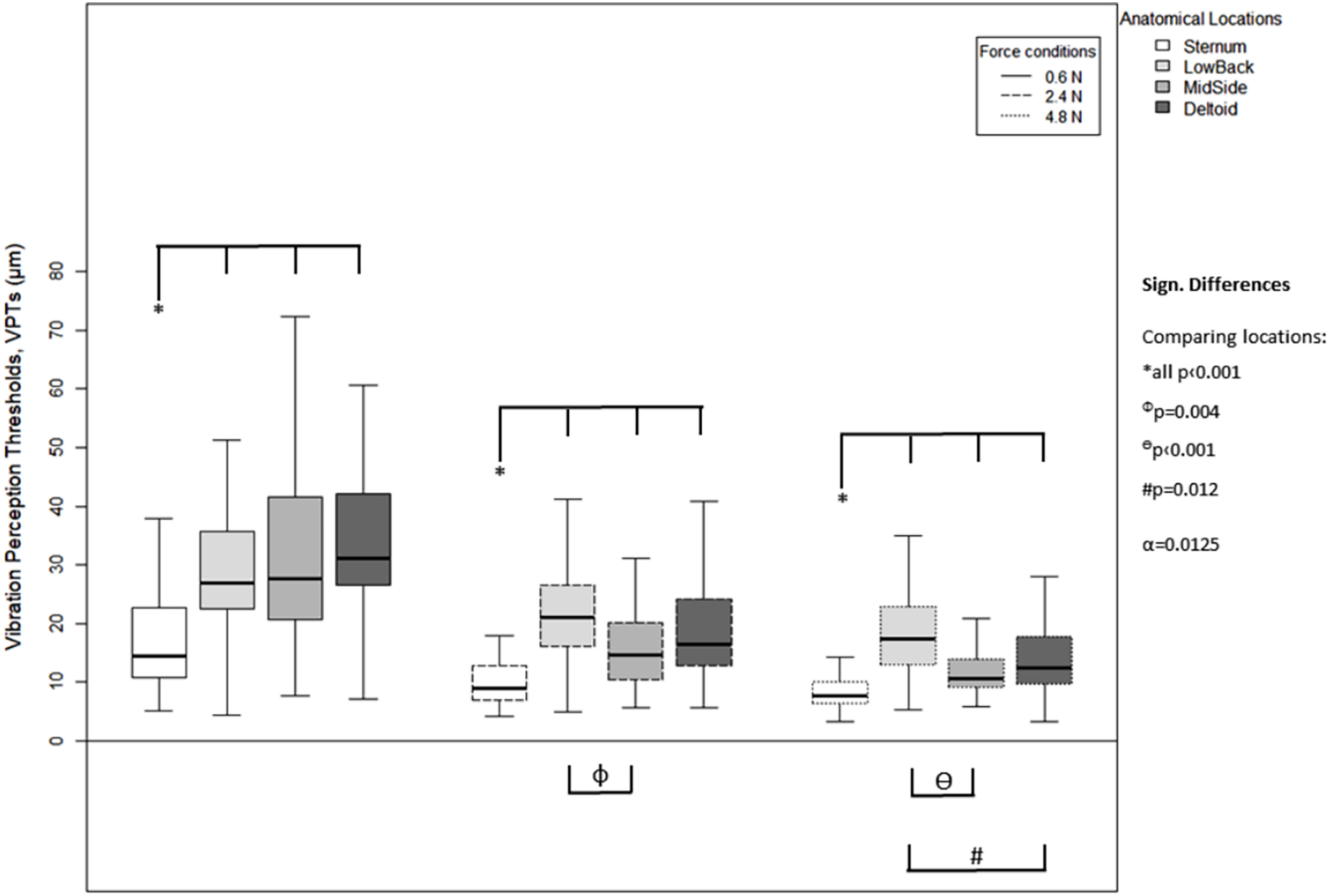
Vibration perception thresholds (VPTs)—comparisons between anatomical locations. Comparison of raw VPT (µm) values between the four anatomical locations (Sternum, LowBack, MidSide, Deltoid) for each force level (0.6, 2.4, 4.8 N, respectively). Inferential statistical tests were performed on logarithmized data (alpha = 0.0125). Asterisks indicate a significant difference between the Sternum compared to the other three locations.

For each of the three force levels, differences between the anatomical locations were evident in the VPT data (Fig. 4). For all force levels, the Sternum exhibited significantly lower VPT thresholds compared to the other three locations (all *p* < 0.001), MidSide exhibited significantly lower VPTs compared to LowBack (for 2.4 N, *p* = 0.004; and 4.8 N, *p* < 0.001), and Deltoid exhibited significantly lower VPTs than Low Back, but only at 4.8 N (*p* = 0.012).

**Figure 5:**
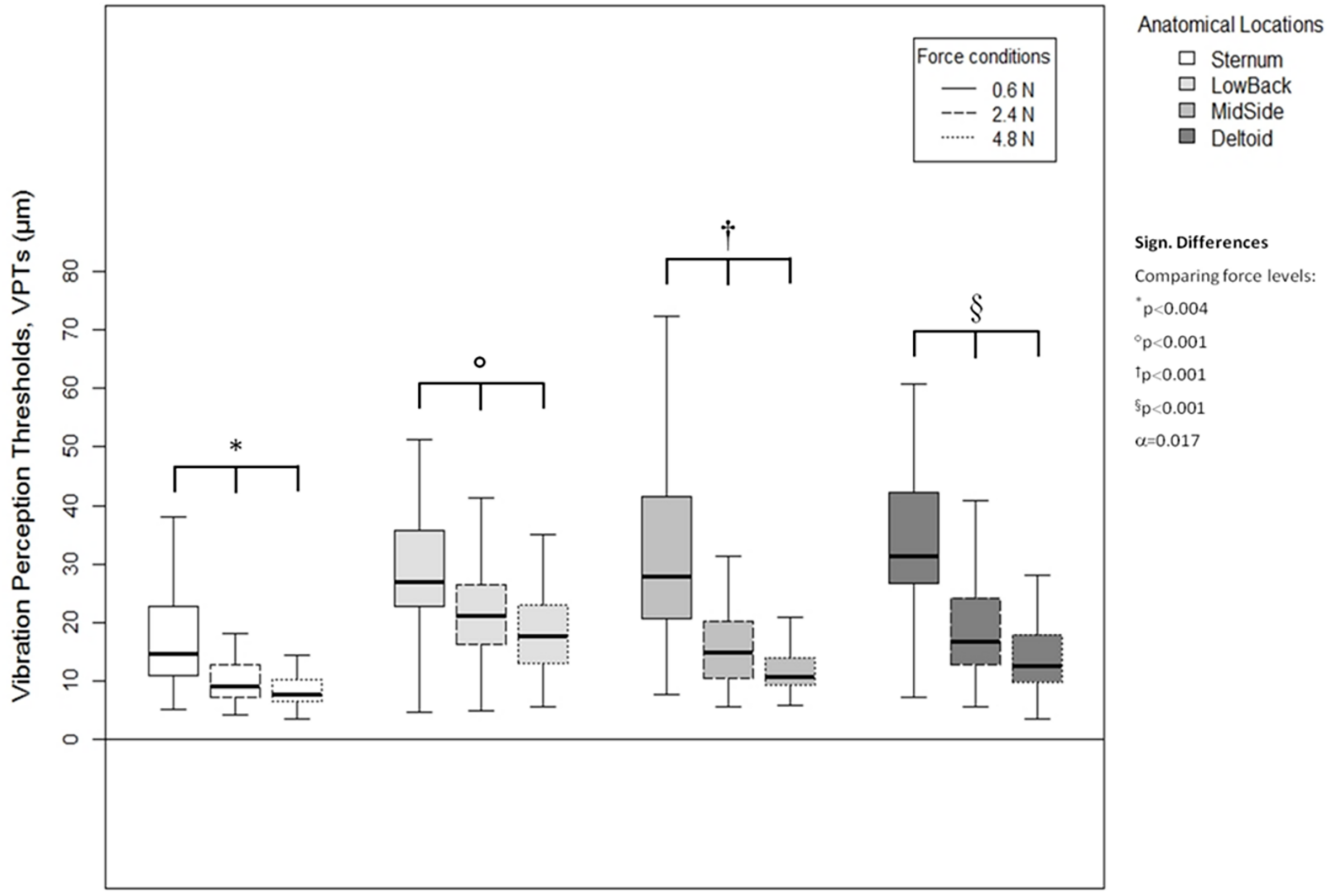
Vibration perception thresholds (VPTs)—comparisons between contactor forces. Comparison of raw VPT (µm) values between the three vertical contactor forces (0.6, 2.4, 4.8 N) for each of the four anatomical locations (Sternum, LowBack, MidSide, Deltoid, respectively). Inferential statistical tests were performed on logarithmized data (alpha = 0.0125).

A higher vertical contact force always corresponded to significantly lower VPTs. These significant differences were true for all three force comparisons (0.6 *vs*. 2.4 N, 0.6 *vs*. 4.8 N, and 2.4 *vs*. 4.8 N) and for all four anatomical locations (Sternum: all *p* < 0.004, LowBack: all *p* < 0.001, MidSide: all *p* < 0.001, and Deltoid: all *p* < 0.001), see Fig. 5.

## Discussion

The present study investigated the influence of different vertical contactor forces (0.6, 2.4, and 4.8 N) on vibration perception thresholds (VPTs, 30 Hz) of human hairy skin at four anatomical locations of the torso in forty individuals. In general, higher contactor forces resulted in lower VPT and thus in improved vibration perception. This was true for all four anatomical locations. Regional differences in vibratory perception were also evident.

### Skin temperatures

Despite significantly increased deltoid temperatures at the post test, the mean difference was only 0.7 °C, which we did not consider relevant. Note that the temperature data stemming from the device with the offset error also resulted in similarly small temperature fluctuations regarding the pre *vs*. post comparison. In earlier work by our group (unpublished data), we were unable to identify a correlation between VPTs and similarly narrow skin temperature fluctuations. Hence, in the present work, we calculated the Spearman correlation between the individual VPT data (for each anatomical location and each force level) and both skin temperature conditions (pre and post), implementing the 18 participants mentioned in the results section. Although rho-values ranged between −0.369 and +0.440 (with 62.5% of the values ranging between −0.200 and +0.200), none of the 24 correlations turned out to be significant (all correlation tests were performed on logarithmized data). This indicates that within such small temperature ranges, lower and higher skin temperatures do not determine lower or higher cutaneous sensitivity, respectively. Additionally, an earlier study found that skin temperature changes of approx. ±5.0 °C induce altered VPTs (Schlee, Sterzing & Milani, 2009)—a magnitude much greater than the differences observed in the current study. Other investigations (Nurse & Nigg, 2001; Germano, Schmidt & Milani, 2016; Schmidt, Germano & Milani, 2017) identifying an effect of skin temperatures on skin sensitivity also implemented clearly larger temperature fluctuations compared to the present study. However, there are other studies that report weak or no effects of cutaneous temperature changes on sensitivity (Gerr & Letz, 1994; Thyagarajan & Dyck, 1994). Based on these considerations, we presume that the small temperature fluctuations we found did not significantly influence VPTs.

### Physiological background

In this section, we aim to briefly discuss what type(s) of mechanoreceptor(s) and corresponding afferents might be responsible for detecting 30 Hz vibrations in human hairy skin of the upper body. As already mentioned, human hairy skin is innervated by five afferent units: SAI (associated with touch domes), SAII (commonly associated with Ruffini corpuscles), and three types of FA units (hair follicle units, field units, and Vater-Pacini corpuscles) (Corniani & Saal, 2020).

Although Vater-Pacini corpuscles exhibit their performance optimum at higher frequencies around 200 Hz (Strzalkowski, Ali & Bent, 2017), several studies (Birznieks et al., 2019; Morioka & Griffin, 2005; Strzalkowski, Ali & Bent, 2017) were able to show afferent discharges or even perceptions at considerably lower frequencies—similar to the present study. Although these investigations examined glabrous skin, we cannot exclude an involvement of Vater-Pacini corpuscles. This is also because Vater-Pacini corpuscles were found in sub-cutaneous tissues, like the periosteum (Sugai et al., 2021). This might play a role for anatomical locations close to bony tissue, like the Sternum or MidSide area in our study.

In addition, an afferent unit known to be highly sensitive to frequencies between 4 and 45 Hz (even up to 150 Hz when using small contactors) resides in the skin: rapidly adapting hair follicle receptors and their afferent channel called “NP _h mid_” (non-Pacini channels in hairy skin at midrange frequencies, likely superficially located within the skin) (Bolanowski, Gescheider & Verrillo, 1994). In general, hair follicle receptors and their afferents have been reported to respond to vibrations ranging from 2–100 Hz (Stuart et al., 2003), whereas others describe target frequencies below about 80 Hz (Mahns et al., 2006). Therefore, it is probable that these afferents contribute to the mediation of the perception of vibratory stimuli as used in this study.

Another potential contributor is the rapidly adapting (Roudaut et al., 2012; Hamalainen, Warren & Gardner, 1985) field unit, also called A-beta field low threshold mechanoreceptors (Aβ field-LTMR), innervated by highly branched axons that form circumferential endings around hair follicles (Handler & Ginty, 2021). Field units are responsive following skin contact stimuli induced by punctuate probes (Macefield, 2005; Hamalainen, Warren & Gardner, 1985). However, field units are less sensitive to pressure pulses (Hamalainen, Warren & Gardner, 1985) but are considerably more active when a stimulus gently strokes across the skin or moves the hair (Handler & Ginty, 2021; Roudaut et al., 2012). Our participants were asked to indicate the presence of vibration stimuli and not the static load of the probe. Hence, from this perspective, it seems uncertain whether or not field units constitute a relevant perceptual contributor in our protocol. However, Ribot-Ciscar, Vedel & Roll (1989) showed that vibrations (20, 40 Hz) simultaneously applied over skin scratching, with phases of scratch application and withdrawal, increased the overall afferent discharge activity of mechanoreceptors in hairy skin (known as masking). Theoretically, the presence of the vibrations in the present study (30 Hz) could have led to a similar effect. Therefore, we cannot entirely disregard a contribution of field units.

Merkel cells, also called touch domes in hairy skin, are located quite superficially, adjacent to hairs in humans. These cells also show afferent discharge activity following skin indentations (Handler & Ginty, 2021). In contrast to field units, however, touch domes were shown to be highly sensitive to vibrations. In mammals, the frequency range of touch domes includes a wide bandwidth from close to zero up to 300 Hz (Handler & Ginty, 2021). Even frequencies as high as 1,500 Hz have been reported (Gottschaldt & Vahle-Hinz, 1981). Hence, a contribution of touch domes appears likely.

Finally, whenever vertical pressure is applied to the skin, this also results in stretch. Following skin indentations and stretch, afferent discharges were found in (I) Ruffini-like corpuscles in glabrous skin (Birznieks et al., 2009), and (II) the previously mentioned hair follicle receptors (Edin & Abbs, 1991; Handler & Ginty, 2021). Please note that Ruffini corpuscles are not believed to play any role in conscious perception (Birznieks et al., 2009). In contrast, a recent investigation addressed a contribution of Ruffini corpuscles and corresponding afferents to human perception (Watkins et al., 2022). However, several aspects need to be considered with respect to their (Watkins et al., 2022) study. First, glabrous skin was investigated. Second, afferents were excited by electrical intraneural microstimulation, an approach clearly different from applying mechanical vibrations to the skin as performed in our study. Third, out of hundreds of Aβ afferents, only 18 SAII afferents resulted in a clear, electrically evoked perception. Fourth, these perceptions were described as large and diffuse pressure, not vibrations (note that the vibrations in our study resulted in a clear and spatially assignable perception, which is clearly different from the participant descriptions from Watkins et al. (2022)). Finally, Watkins et al. (2022) describe that earlier investigations found highly inconsistent outcomes regarding whether or not activity of Ruffini corpuscles induce perceptions. For these reasons, we consider the involvement of Ruffini corpuscles in vibration perception as performed in our study to be rather unlikely.

In summary, our data indicate that two single mechanical stimuli (*e.g*., varying static indentations and simultaneous vibrations as employed in our study) activate several receptors and afferents to varying degrees. This was also described by Handler & Ginty (2021), who stated that such stimuli result in complex central integration processes finally leading to perception. For our experiments, we can only speculate that vibration perception is based on the interlinked activity of Vater-Pacini corpuscles, hair follicle receptors (NP _h mid_ and possibly field units), and touch domes including their afferent axons, respectively.

### VPT comparisons between sexes

For each anatomical location and for each force level, there were no inferentially statistical VPT differences between females and males. Considering not only mean differences but holistic data (*e.g*., ranges or boxplots), this finding was confirmed with these descriptive measures. Additionally, calculated effect sizes were generally small. For Cohen’s D, nine out of ten values were small (<0.420) and one medium (0.580) (Cohen, 1988). For the two Vargha and Delaney’s A effect sizes, both values were small (Vargha & Delaney, 2000). Although the small effect sizes seem to support the notion of absent sex differences, they may also be explained by the large variability of the data due to the limited number of participants. Hence, we cannot entirely disregard differences. Overall, however, we think it is likely that there are no sex differences in our VPT data.

This notion is also supported by the fact that the absence of sex differences in VPT data (especially for young participants like in the present study (age range 20–27 years)) is commonly reported in the literature (*e.g*., Deshpande et al., 2008; Germano et al., 2016; Halonen, 1986; Schlee, Sterzing & Milani, 2009; Schmidt et al., 2020; Schmidt, Germano & Milani, 2018, 2017). This would also facilitate the selection of participants, making it unnecessary to analyze corresponding data separately according to sex.

### Differences between anatomical locations

In general, our absolute VPTs (raw data) appear to be comparable to other studies. Although Mahns et al. (2006) found clearly higher thresholds at the forearm (149 µm at 20 Hz), it is noteworthy to mention several possible reasons for this discrepancy. First, they used a smaller probe resulting in elevated thresholds due to spatial summation effects. Second, Mahns et al. (2006) did not mention concrete vertical contactor forces. Third, the forearm is a region clearly different from the regions examined in our study. An early investigation by Wilska (1954) found similar VPTs at a testing frequency of 50 Hz and included anatomical regions close to ours. However, Wilska (1954) also used a smaller contactor, did not report contactor forces, and investigated only one male participant.

With respect to the present investigation, the Sternum was the most sensitive region, regardless of the force level applied. In terms of afferent innervation densities (valid for A-beta fibers only), the chest, abdomen, and back were shown to exhibit quite uniform values around 8.9–9 units/cm^2^ (Corniani & Saal, 2020). However, previous studies have shown that the human torso is not uniformly sensitive with respect to tactile stimulation (Schmidt et al., 2020; Stronks, Parker & Barnes, 2016; Wilska, 1954). Wilska (1954) implemented vibrations of varying frequencies (50, 100, 200, 400, and 800 Hz) and found similar regional vibratory sensitivity differences among the torso (Sternum region more sensitive than LowBack region, for example). Stronks, Parker & Barnes (2016) investigated the two-point discrimination and just noticeable differences at the lower back by implementing vibrating coin motors. In their work, they mention studies confirming that the back is not uniformly sensitive with respect to these measures using static or vibratory stimuli. Finally, Schmidt et al. (2020) investigated vibratory sensitivity with various contactor dimensions and two frequencies (30, 200 Hz). They found regional differences within the upper body, especially for the larger contactor. These studies support our findings of non-uniform torso sensibility.

Possible reasons for different cutaneous sensitivity across different body regions are different receptor densities (Meh & Denislic, 1995; Stuart et al., 2003). However, this may not fully account for varying sensitivities. For example, in the hairy skin of the torso, hair follicle receptors associated with the NP _h mid_ channel are bound to hair follicles. Similarly, touch domes are located adjacent to hair in humans (Handler & Ginty, 2021), which would imply a positive correlation between the density of hair follicles and the number of touch domes. Indeed, Corniani & Saal (2020) found a strong correlation between innervation densities and hair follicle densities. Interestingly, an earlier investigation found a hair follicle density between similar regions that tends to contradict our findings (from lower to higher density: thorax—back—upper arm) (Otberg et al., 2004). Indeed, Verrillo & Chamberlain (1972) conclude that cutaneous sensitivity is not necessarily determined by the neural density, but rather by the number of sensory afferent units activated by a stimulus. This is particularly present when the stimulus is allowed to propagate freely, for example with no contactor surround, as in our study. This then activates other more distant afferents within the skin or adjacent bone material. Lamoré & Keemink (1988) also argue different unit densities (not only receptors) are responsible for different vibratory sensitivity in different body regions.

In this context, it is interesting to note two aspects. First, one specific plantar tactile stimulus, such as a certain vibration or vertical pressure, is capable of simultaneously activating different cutaneous receptor classes (Lowrey, Strzalkowski & Bent, 2013). It is highly likely that this aspect also occurred in our protocol stimulating hairy skin and that this was region-dependent. Second, it is known that Vater-Pacini corpuscles are not only present in the skin, but also in subcutaneous tissues like the periosteum (Sugai et al., 2021). This would explain why we found a higher vibration sensitivity for the Sternum, a location closer to bone tissue, than all the other regions investigated in our study. Furthermore, Birznieks et al. (2019) discuss that there are interactions between different afferent inputs. The vast majority of rapidly adapting neurons in the somatosensory cortex S1 area show convergent inputs from different afferent classes, affecting actual perception (Birznieks et al., 2019). This might help to explain our findings of different sensibilities across the torso, especially with regard to the Sternum. From all locations examined in our study, the skin at the Sternum is particularly close to bone material. Vater-Pacini corpuscles are present in the periosteum of bones (Sugai et al., 2021) and we cannot disregard their contribution toward detecting 30 Hz vibrations (in addition to the other cutaneous receptors at the Sternum). Consequently, the concept that a vibration might activate multiple receptors (particularly at the Sternum) to facilitate a perception appears possible. Ultimately, the so-called myelinated innervation density may also be a factor. Although related to rodent glabrous skin, Meissner corpuscles in one rodent were innervated by a higher number of axons at the forepaw compared to the hind paw (Walcher et al., 2018). It would be interesting to examine whether such mechanisms also occur in human hairy skin, which would constitute another explanation for the regional sensitivity differences we found.

In addition to peripheral sensory and afferent factors, it was also proposed that attention plays an important role in terms of perceiving tactile stimuli (Macefield, 2005). An interesting investigation by Mirams et al. (2012) mentioned that focusing on exteroceptive information affects perceptual effects, *e.g*., possibly reducing sensory noise. Mirams et al. (2012) also cite work confirming that attention enhances tactile perception by amplifying neural responses in the somatosensory cortex. The fact that not only peripheral factors (mechanoreceptors and their first-order neuronal afferents) and the spinal cord are responsible for perception also becomes evident in another investigation: Gallace & Spence (2008) postulated that the awareness of tactile information is also affected by higher-order, associative (likely multisensory) brain areas, such as the posterior parietal cortex. Finally, other modalities (*e.g*., vision) may also affect the perception of tactile stimuli, yet at the unisensory somatosensory cortex level (Gallace & Spence, 2008). Within the present study, however, it remains unclear how these factors may have changed during our protocol/between anatomical locations.

### Differences between vertical contactor forces

At all anatomical locations, we confirmed that vibratory sensitivity improved significantly with increasing vertical contactor forces. Some studies report no significant effect of contactor forces on vibration perception (Gregg, 1951; Hagander et al., 2000). Compared to our results, this discrepancy may be explained by the following aspects. First, although Gregg (1951) states that the effect of the contactor pressure is “negligible”, the author did not provide detailed information of relevant co-parameters associated with these measurements. In this regard, there is a lack of information regarding implemented body regions, the used contactor force/pressure levels, and contactor size(s). Furthermore, Gregg (1951) mentioned exceptions: exerting “too little pressure” and “excessive (almost painful) pressure” might indeed affect vibration perception. Again, Gregg (1951) did not provide concrete information about this. A second aspect is related to the study conducted by Hagander et al. (2000). In their study, they stated that pressure-induced differences are “clinically negligible”. However, this was based on the limited sensitivity of their vibrating device (100 µm). This is clearly inferior to the capabilities of the device used in our study and may help explain differences. Finally, considering glabrous skin areas, our findings coincide with numerous other studies identifying an effect of vertical contactor forces on VPTs (*e.g*., Lowenthal, Derek & Hockaday, 1987; Cassella, Ashford & Kavanagh-Sharp, 2000; Gu & Griffin, 2012; Zippenfennig, Wynands & Milani, 2021). Similarly, increased airpuff intensities (forces) applied to the dorsal hand (hairy skin) resulted in improved cutaneous sensitivity parameters (Hamalainen, Warren & Gardner, 1985). Increased activity of unmyelinated afferents following higher indentation forces (pressure stimuli) was also shown (Vallbo, Olausson & Wessberg, 1999), which would infer the possibility of perceiving pain. Although we did not assess the presence of pain by using a visual analogue scale, our participants did not report any pain during the measurements.

Lowenthal, Derek & Hockaday (1987) did not provide a concrete explanation for improved sensitivity following increased vertical contactor forces, but argued that more receptors might be triggered. As mentioned previously, stimulating a greater quantity of receptors may not fully explain improved sensitivity. The number of activated afferent units is more decisive. The indentation amplitude of the probe also increased with increasing vertical contactor forces. Consequently, more area of the skin was affected, especially because the contactor in our experiments was “free-standing” (no surround). Prior to the vibratory stimulus itself, the vertical indentation causes a stretch and increased tension of the skin. Once the vibration occurs, it is conceivable that with higher contact forces, vibration propagates more easily within the larger (pre-stretched) skin area, similar to spatial summation effects. Although glabrous skin is stiffer than hairy skin, meaning that skin displacements produce larger forces in glabrous compared to hairy skin (Pubols, 1982), we believe that this effect is still present to a substantial extent in our experiments.

The above-mentioned argument means that with increasing vertical contactor forces, more afferent units would be more intensively activated leading to improved vibratory perception. Although based on a neurophysiological animal study, Iggo & Muir (1969) were able to demonstrate that during dynamic and static indentations, the firing frequency of single slowly adapting touch receptors/single afferent units increased for large indentations/vertical forces compared to small indentations/vertical contactor forces. A similar behavior also seems to be present considering fast adapting cutaneous mechanoreceptors/afferents. Phillips, Johansson & Johnson (1992) applied a matrix of various dots with varying spacings on human fingerpad skin. For a wide range of dot spacings, they found that the lowest vertical force level (0.4 N) was associated with a lower afferent discharge rate compared to the two higher vertical force levels (0.6 and 1.0 N). A microneurographic study performed at a cat’s central foot pad (hindlimb) also found that rapidly adapting receptors exhibited a higher discharge rate at increased pressure/force levels compared to low pressure/force levels (Jaenig, Schmidt & Zimmermann, 1968). Finally, it was also shown for human hairy skin (dorsal hand) that a specific fast adapting unit exhibited elevated afferent discharge rates with higher strain velocities following a stretch stimulus (Edin, 2004). Therefore, we believe that it is possible in our experiments that the vertical contactor force *per se* enhanced afferent responses of single units. This might then lead to an increased likelihood of conscious stimulus perception. In an earlier study, we were able to demonstrate that spatial summation likely occurs during 30 Hz vibrations in the human hairy skin of the torso (Schmidt et al., 2020). This clearly addresses the aspect of activating more afferent units by increased contactor forces. This was evident by improved vibrotactile perception with an increasing size of the contactor. In another study, Hamalainen, Warren & Gardner (1985), showed that an increased number of stimulus points enhances tactile sensation to a much greater extent on hairy than on glabrous skin. In addition, one single stimulus is capable of activating several receptor types and/or afferent units (Birznieks et al., 2019; Gardner & Martin, 2000; Lowrey, Strzalkowski & Bent, 2013). This appears especially true for the Sternum location, where bone material (containing Vater-Pacini corpuscles) is located in close vicinity. Although investigating glabrous skin, it has also been shown that Merkel discs and Meissner corpuscles can act as a unit (Abraira & Ginty, 2013). Hence, similar effects with receptors in hairy skin appear likely, which might explain the sensitivity properties we found. Considering the above-mentioned aspects and that increased contactor forces enlarge and intensify the mechanical stimulation of the skin, our results and arguments appear plausible. This suggests that the contactor activates more than one afferent, especially at higher forces, which contribute to the overall cortical response and perception.

In conclusion, the present study demonstrated that increased vertical contactor force improves vibrotactile perception at 30 Hz in hairy skin of the torso, regardless of the anatomical location. This may be due to a greater number of (different) afferent units being activated. Furthermore, we found that vibrotactile sensitivity was particularly high at the sternum location compared to the other three regions, regardless of the force level applied. The reasons for this may also be related to the activity pattern of different afferent units. Ultimately, no sex-specific differences of vibration perception were found at any regions or force levels. The present findings complement the understanding of vibrotactile sensitivity of hairy skin, including several factors that should be considered in future research, such as the vertical contactor force or regional sensitivity differences. This could be important for the development of vibrotactile devices or wests, for example. In this regard, warning functions require a fast and low-threshold signal perception. Similarly, certain identifiable patterns induced by vibrating arrays might serve as spatial information when walking, for example. Our results suggest that locations in close proximity to bony structures and contact forces that are slightly higher rather than too low constitute important factors.

This study also has its limitations. First, only one frequency (30 Hz) was considered. It would be interesting to see if similar effects would also be present when considering a wider frequency range. In this context, it was shown that the effect of contactor forces on plantar VPTs is not uniform across lower (30 Hz) and higher (200 Hz) frequencies (Zippenfennig, Wynands & Milani, 2021). Second, following the discussion about the effects of the indenting contactor on skin stretch/deformation, it would also be interesting to consider other contactor sizes. Particularly flat and larger-scaled contactors might mimic everyday situations to a better extent (wearing clothes, for example) and might provide more information for wearable devices. Third, the optimal stimulus to elicit a response in hair follicle receptors is hair motion by bending the hair shaft (Hamalainen, Warren & Gardner, 1985). Hence, stimulating the hair follicle receptors using a rigid contactor indenting into the skin might not be the optimal method of receptor activation. Therefore, more precise conclusions about the underlying receptors and afferents may be formed if these three limitations are overcome.

## Conclusions

This study examined the effects of vertical contactor forces (0.6, 2.4, and 4.8 N) and different anatomical locations (sternum, deltoid/shoulder, lower back, middle lateral torso side) on vibration perception (at 30 Hz) in forty young and healthy individuals. Higher contactor forces generally corresponded to lower VPTs constituting improved vibration perception. Furthermore, the sternum was the most sensitive region. The reasons for these findings may lay in a varying number and activation patterns of afferents activated under the different conditions. Our findings complement the understanding of vibrotactile sensitivity in hairy skin and may offer implications when developing vibrotactile devices or clothing/textiles, for example.

## Supplemental Information

10.7717/peerj.15952/supp-1 Supplemental Information 1 Raw data of anthropometrics. Mean ± standard deviation (SD) anthropometric data for all participants, females, and males.

10.7717/peerj.15952/supp-2 Supplemental Information 2 Raw data of vibration perception thresholds (VPTs). Mean vibration perception thresholds (VPTs) are presented for each force level and anatomical location. Data is shown for all 40 participants.

## Additional Information and Declarations

### Data Availability

The following information was supplied regarding data availability: The raw data is available in the Supplemental Files.
